# Retrospective study of necrotizing fasciitis and characterization of its associated Methicillin-resistant *Staphylococcus aureus *in Taiwan

**DOI:** 10.1186/1471-2334-11-297

**Published:** 2011-10-31

**Authors:** Chih-Hsuan Changchien, Ying-Ying Chen, Shu-Wun Chen, Wan-Lin Chen, Jwu-Guh Tsay, Chishih Chu

**Affiliations:** 1 Department of Microbiology, Immunology, and Biopharmaceuticals, National Chiayi University, No 300, University Road, Chiayi, 60004, Taiwan, ROC; 2 Department of Plastic and Reconstructive Surgery, Chiayi Christian Hospital, 539 Jhongsiao Rd. Chiayi City, 60002, Taiwan, ROC; 3 Department of Nutrition and Health Science, Toko University, No.51, Sec. 2, Syuefu Rd., Puzih City, Chiayi County 61363, Taiwan, ROC

## Abstract

**Background:**

Methicillin-resistant *Staphylococcus aureus *(MRSA) has emerged as a prevalent pathogen of necrotizing fasciitis (NF) in Taiwan. A four-year NF cases and clinical and genetic differences between hospital acquired (HA)- and community-acquired (CA)-MRSA infection and isolates were investigated.

**Methods:**

A retrospective study of 247 NF cases in 2004-2008 and antimicrobial susceptibilities, staphylococcal chromosomal cassette *mec *(SCC*mec*) types, pulsed field gel electrophoresis (PFGE) patterns, virulence factors, and multilocus sequence typing (MLST) of 16 NF-associated MRSA in 2008 were also evaluated.

**Results:**

In 247 cases, 42 microbial species were identified. *S. aureus *was the major prevalent pathogen and MRSA accounted for 19.8% of NF cases. Most patients had many coexisting medical conditions, including diabetes mellitus, followed by hypertension, chronic azotemia and chronic hepatic disease in order of decreasing prevalence. Patients with MRSA infection tended to have more severe clinical outcomes in terms of amputation rate (p < 0.05) and reconstruction rate (p = 0.001) than those with methicillin-sensitive *S. aureus *or non-*S. aureus *infection. NF patients infected by HA-MRSA had a significantly higher amputation rate, comorbidity, C-reactive protein level, and involvement of lower extremity than those infected by CA-MRSA. In addition to over 90% of MRSA resistant to erythromycin and clindamycin, HA-MRSA was more resistant than CA-MRSA to trimethoprim-sulfamethoxazole (45.8% *vs*. 4%). ST59/pulsotype C/SCC*mec *IV and ST239/pulsotype A/SCC*mec *III isolates were the most prevalent CA- and HA-MRSA, respectively in 16 isolates obtained in 2008. In contrast to the gene for γ-hemolysin found in all MRSA, the gene for Panton-Valentine leukocidin was only identified in ST59 MRSA isolates. Other three virulence factors TSST-1, ETA, and ETB were occasionally identified in MRSA isolates tested.

**Conclusion:**

NF patients with MRSA infection, especially HA-MRSA infection, had more severe clinical outcomes than those infected by other microbial. The prevalent NF-associated MRSA clones in Taiwan differed distinctly from the most predominant NF-associated USA300 CA-MRSA clone in the USA. Initial empiric antimicrobials with a broad coverage for MRSA should be considered in the treatment of NF patients in an endemic area.

## Background

Necrotizing fasciitis (NF) is a rare and life-threatening infection that involves deep soft tissue and is characterized by widespread fascial necrosis, with a mortality of 25.3-73% [1-3]. Necrotizing fasciitis typically is polymicrobial infection caused by aerobic and anaerobic organism that participate in synergistic infection; whereas Group A *Streptococcus*, *Clostridium *spp., *Vibrio *spp. and *Klebsiella *spp. have been the cause of monomicrobial infection. Although *Staphylococcus aureus *is the most prevalent pathogen in both hospitals and communities and MRSA has become a common isolate associated with skin and soft tissue infections globally over the past few years, monomicrobial MRSA necrotizing fasciitis has been reported only in a few studies [4-6]. In Taiwan, MRSA accounts for 53-83% of *S. aureus *isolates in most major hospitals [7,8]. Community-acquired MRSA (CA-MRSA) has become increasingly endemic, accounting for 74% and 28% of all MRSA in two studies that were performed in northern and central Taiwan, respectively [9]. Recently, MRSA has been identified as an important cause of necrotizing fasciitis in Taiwan [10].

Several virulence factors are reportedly associated with the pathogenicity of skin and soft tissue infections. Panton-Valentine leukocidin (PVL), a pore-forming exotoxin, and γ-hemolysin (Hlg) are associated with pus-forming diseases. CA-MRSA isolates with the PVL genes generally cause skin and soft tissue infections and necrotizing fasciitis [4,11]. Toxic shock syndrome toxin 1 (TSST-1) can cause in part a desquamative skin rash and toxemic syndrome [12]. Exfoliatin A (ETA) and exfoliatin B (ETB) are associated with numerous blistering skin diseases and staphylococcal scalded-skin syndrome [13]. The variable clinical presentations of *S. aureus *skin and soft tissue infections are believed to be associated with the particular combinations of virulence factors and regulatory genes given an appropriate genetic background [14].

In staphylococcal species, low-affinity penicillin-binding protein 2a, which is responsible for methicillin resistance, is produced by the *mecA *gene in an exogenous mobile staphylococcal chromosomal cassette *mec *(SCC*mec*) [15,16]. Hence a methicillin-susceptible *S. aureus *(MSSA) isolate may acquire the SCC*mec *elements and thereby become an MRSA isolate [17]. Based on variations in the *ccr *operon and *mec *complexes of the SCC*mec*, at least seven SCC*mec *types have been identified as having some clinical implications [18]. The SCC*mec *IV element (~24 kb) is most commonly identified in CA-MRSA isolates, whereas the SCC*mec *II and III elements are often found in HA-MRSA isolates [19,20].

The management of NF absolutely depends on the prompt administering of suitable antibiotics and adequate surgical debridement. Increasing prevalence of MRSA, the choice of appropriate antibiotics for NF patients in the community must now take into account the increasing global prevalence of MRSA and the patterns of antimicrobial susceptibility [21]. Therefore, a study population of 247 NF cases in 2004-2008 and their associated MRSA isolates were investigated to understand the epidemiological or clinical effects of MRSA on NF cases and clinical and genetic differences between CA- and HA-MRSA. Further, antimicrobial susceptibility, SCC*mec *types, pulsed field gel electrophoresis (PFGE) patterns, virulence factors, and MLST types of MRSA strains that were isolated from 16 NF patients in 2008 were examined.

## Methods

### Study population

A total of 247 NF patients were consecutively hospitalized and surgically treated at the 1000-bed Chiayi Christian Hospital in southern Taiwan from December 2004 to November 2008. A patient was included in the study if the postoperative diagnosis in his or her surgical report was of necrotizing fasciitis that this diagnosis was verified by pathological examination, as determined by an electronic search and a discharge diagnosis of necrotizing fasciitis. Necrotizing fasciitis is defined by the followings; the presence of grayish necrotic fascia; a demonstrated lack of resistance of muscular fascia that was freely dissected using a blunt instrument along the normal adherent tissue planes; the presence of foul-smelling "dish-water" pus; and/or, extensive necrosis of the skin and subcutaneous tissues [3]. Electronic and paper medical records were reviewed for clinical variables that included age, gender, infection site, and underlying chronic conditions such as diabetes mellitus, hypertension, chronic liver disease, coronary artery disease, chronic renal insufficiency, chronic obstructive pulmonary disease, and malignancy. Empiric antibiotics that were used in initial treatment, the number of surgical debridements and reconstructions undergone, amputation, period of hospitalization, in-hospital mortality rate, laboratory findings at the time of admission, and clinical outcomes in each NF case were also evaluated. A surgeon and a nurse practitioner of plastic surgery collected the data. These data were then compared across four distinct groups with different etiologic pathogens (non-*S. aureus*, MSSA, CA-MRSA, and HA-MRSA) to determine whether the clinical variables were associated in any way with MRSA necrotizing fasciitis.

### Antimicrobial susceptibility test

The susceptibility of *S. aureus *isolates to antimicrobials clindamycin, erythromycin, fucidin, linezolid, oxacillin, teicoplanin, trimethoprim-sulfamethoxazole, and vancomycin was performed by using disc diffusion and the guideline of Clinical and Laboratory Standards Institute (CLSI) [22]. Isolates with zones of inhibition that fell into the category of intermediate susceptibility to a given antibiotic were regarded as susceptible isolates.

### Characterization of NF-associated MRSA isolates

A total of 16 specimens were obtained from wound cultures of NF patients upon initial surgical debridement or from blood cultures of NF patients who had visited hospital between January 2008 and November 2008. Blood cultures were processed in a VITEK 2 GP system (BioMerieux) in the hospital laboratory. Bacteria were firstly analyzed by coagulase testing and Gram staining. *S. aureus *was identified by the polymerase chain reaction (PCR) amplification of *S. aureus*-specific *clfA*, 16S rDNA, and *nuc *genes [23,24]. The *S. aureus *isolates BCRC 10781 and BCRC 15211 were used as methicillin-susceptible and resistant reference strains, respectively. MRSA isolates were separated into HA- or CA-MRSA using the following definition. HA-MRSA is an MRSA isolate that is obtained from patients who stay more than 48 hours after admission to hospital, or who have established risk factors, such as a history of HA-MRSA isolation, hospitalization, surgery, dialysis or admission to a nursing home, skilled nursing facility or hospice in the past year or who have a permanent indwelling catheter or medical device that passes through the skin into the body [21]. In contrast, a CA-MRSA isolate is an MRSA isolate that is obtained from a patient within 48 hours of admission to the hospital and without the above conditions for HA-MRSA.

### Genetic analysis by pulsed field gel electrophoresis (PFGE) and MLST

The genotype of each isolate was identified by *Sma*I-digested PFGE analysis using a method described elsewhere [25]. Briefly, whole-cell embedding agarose plugs were digested using restriction endonuclease *Sma*I. The DNA fragments were resolved using a CHEF DR-III apparatus (Bio-Rad). The standard size marker was the MRSA isolate BCRC 15211. Isolates are regarded as closely related if they differ by no more than three fragments; as possibly related if they differ by four to six fragments, and unrelated if they differ by at least seven fragments [26]. MLST types of 16 CA- and HA-MRSA isolates were identified using the method that was described by Enright [27] and by analysis of MLST databases (http://saureus.mlst.net/).

### PCR identification of SCC*mec *types and genes for virulence factors PVL, Hlg, TSST-1, ETA, and ETB

SCC*mec *types I-IV were identified by multiplex PCR amplification of SCC*mec *regions [16,28]. If not groupable into types I-IV, isolates were grouped into SCC*mec *type V or VT (or VII) by the PCR detection of *ccrC *(*ccr5*) homologues [29-31]. Genes for virulence factors PVL, Hlg, TSST-1, ETA, and ETB were identified by simplex and multiplex PCR amplifications using primers described elsewhere [14,32].

### Statistical analysis

Categorical variables were presented as frequencies and percentages, and continuous variables were presented as means and standard deviations. The chi-square test or Fisher's exact test were adopted to compare categorical data, as appropriate. Continuous data were obtained by analysis of variance to evaluate the differences between the groups, and a post hoc comparison was conducted using the Tukey honestly-significant difference test. All reported P values were two-tailed, and a P value of 0.05 indicated statistical significance. All analyses were performed using SPSS software.

## Results

### Demographics and clinical variables

The dataset from the specified four-year period included a total of 247 cases of necrotizing fasciitis, with a 19.8% prevalence of MRSA as the causative organism of the infection. The mean age, gender, mean number of surgeries and mortality rate of the NF patients did not vary much with the cause of the infection by non-*S. aureus*, MSSA, CA-MRSA, or HA-MRSA (Table 1). Most patients in this investigation had multiple coexisting medical conditions, including diabetes mellitus (62.8%), hypertension (30.1%), chronic azotemia (28.2%) and chronic hepatic disease (14.6%). The incidence of peripheral artery occlusive disease and malignancy was extremely low in all four groups, which did not discernibly differ in this respect. Patients who were infected by HA-MRSA exhibited significantly more comorbidity and a higher incidence of chronic azotemia than the other three groups (p < 0.05). Regardless of infecting species, the lower extremities were the most common infection sites (p < 0.05). NF patients of two *Vibrio *monomicrobial, three non-*S. aureus *polymicrobial, and one HA-MRSA died. No death occurred in the MSSA and CA-MRSA groups. Two CA-MRSA, one HA-MRSA, and four non-*S. aureus *NF patients presented a systolic blood pressure of <90 mm Hg when they arrived in the emergency room. As determined by univariate analysis, patients who were infected by CA-MRSA had a lower C-reactive protein level than those infected by HA-MRSA, MSSA, or non*-S. aureus*. Body temperature, leukocyte count and platelet count at the time of admission to hospital were similar among the four groups of NF patients. Patients who were infected with MRSA tended to have more severe clinical outcomes in terms of amputation rate (p < 0.05) and reconstruction rate (p = 0.001) than those with MSSA or non-*S. aureus *infection. Moreover, NF patients with HA-MRSA infection had a higher rate of amputation on admission (29.2%) than those with CA-MRSA infection (8%) (p < 0.05).

**Table 1:** Clinical characteristics of 247 NF cases and antimicrobial resistance of CA- and HA-MRSA isolates

Characteristic	Non-*S. aureus* (n = 156)	MSSA (n = 42)	CA-MRSA (n = 25)	HA-MRSA (n = 24)	p-value
Age (mean, yr)^§^ (years)	59.1 ± 13.0	56.0 ± 17.9	53.8 ± 14.3	57.4 ± 15.3	0.262
Male (n, %)	107 (68.6)	27 (64.3)	13 (52)	17 (70.8)	0.399
Comorbidities (n, %)^†^	1.8 (1.2)	1.9 (1.5)	1.4 (1.1)	2.9 (1.5)	<0.001***
Hospitalization (mean, day)^§^	17.2 ± 10.6	15 ± 8.6	13.2 ± 8.3	20.3 ± 8.1	0.052
Underlying chronic disease					
Diabetes mellitus (n, %)	95 (60.9)	25 (59.5)	15 (60.0)	20 (83.3)	0.183
Hypertension (n, %)	59 (37.8)	17 (40.5)	7 (28.0)	11 (45.8)	0.616
Chronic hepatic disease (n, %)	19 (12.2)	8 (19)	4 (16)	5 (20.8)	0.451
Malignancy (n, %)	6 (3.8)	2 (4.8)	0 (0)	0 (0)	0.765
Obesity (n, %)	17 (10.9)	3 (7.1)	0 (0)	1 (4.2)	0.300
Pulmonary disease (n, %)	11 (7.1)	3 (7.1)	0 (0)	2 (8.3)	0.600
Chronic azotemia (n, %)	26 (16.7)	6 (14.3)	2 (8)	10 (41.7)	0.019*
Operative procedure (mean, n)	2.1 ± 1.1	1.9 ± 0.6	2.1 ± 0.9	2.4 ± 1.5	0.237
Reconstructive surgery (n, %)	15 (9.6)	2 (4.8)	8 (32)	7 (29.2)	<0.001***
Amputation (n, %)	18 (11.5)	2 (4.8)	2 (8)	7 (29.2)	0.037*
Mortality (n, %)	5 (3.2)	0 (0)	0 (0)	1 (4.2)	0.542
Site of infection					
Upper extremity (n, %)	18 (11.5)	8 (19)	4 (16)	1 (4.2)	0.315
Lower extremity (n, %)	103 (66)	25 (59.5)	17 (68)	20 (83.3)	0.005**
Head and neck (n, %)	1 (0.6)	4 (9.5)	2 (8)	1 (4.2)	0.014*
Trunk (n, %)	34 (21.8)	5 (11.9)	2 (8)	2 (8.3)	0.118
Body temperature (°C) (mean)^§^	36.9 ± 0.8	36.9 ± 0.5	36.8 ± 0.5	37.1 ± 0.7	0.624
Shock (n, %)	4 (2.6)	0 (0)	2 (8)	1 (4.2)	0.170
Leukocyte count (mean cells/mm^3^)^§^	13.9 ± 7.4	14.1 ± 5.1	12.9 ± 5.5	15.1 ± 7.7	0.752
Platelet count (mean, per mm^3^)	248.3 ± 107.9	263.2 ± 96.9	290.5 ± 120.2	293.9 ± 99.5	0.101
C-reactive protein (mean, mg/dl)^‡^	15.8 ± 13.2	12.2 ± 11.8	10.4 ± 10.6	25.5 ± 14.8	0.005**
					
Antimicrobials resistance			CA-MRSA	HA-MRSA	
Clindamycin (n, %)			23 (92.0)	23 (95.8)	0.576
Erythromycin (n, %)			23 (92.0)	23 (95.8)	0.576
Fucidic acid (n, %)			3 (12.0)	3 (12.5)	0.957
Trimethoprim-sulfamethoxazole (n, %)			1 (4.0)	11 (45.8)	<0.001***

^§ ^Data represents as mean ± standard deviation or number of cases (prevalence, %).

^† ^Post hot comparison using Tukey mothod: Non-*S. aureus *< HA-MRSA, p = 0.001; MSSA < HA-MRSA, p = 0.02; CA-MRSA < HA-MRSA, p < 0.001.

^‡ ^Post hot comparison using Tukey mothod: Non-*S. aureus *< HA-MRSA, p = 0.045; MSSA < HA-MRSA, p = 0.008; CA-MRSA < HA-MRSA, p = 0.007.

* indicates significant differences among four groups (p < 0.05); ** and *** indicates highly significant among four groups with p < 0.01 and p < 0.001 respectively.

### Microbial associated with NF

**Figure 1:**
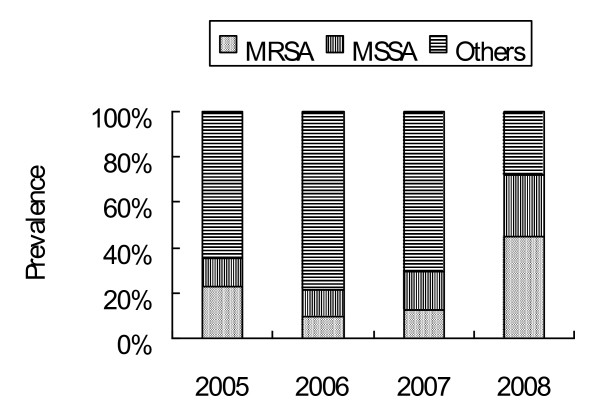
**Prevalence of MRSA and MSSA associated with 247 NF cases in 2005 to 2008**.

From the initial wounds of the 247 NF patients or from blood culture, 34 aerobic bacterial species, seven anaerobic bacterial species and one fungal species were identified (Table 2). The prevalence of polymicrobial and monomicrobial infections was 55.5 and 44.5, respectively. An average of 2.77 microbial species was identified in a polymicrobial infection. The major bacterial species were *Staphylococcus *spp., followed by *Streptococcus *spp., *Enterococcus *spp., *Peptostrep *spp., *Bacteroides *spp., *Prevotella *spp., *Klebsiella *spp., and *E. coli*. Generally, most NF cases were polymicrobial infections. However, the most monomicrobial infections for NF cases were caused by *Staphylococcus *spp. (57.2%) and *Vibrio *spp. (9%). The prevalence of both MRSA and MSSA necrotizing fasciitis increased gradually from 2006 to 2008, and MRSA was the major pathogenic cause of necrotizing fasciitis in 2008 (Figure 1).

**Table 2:** The number of microbial species associated with NF

Organism	Monomicrobial			Polymicrobial			Total (%)
		
	Number	A,%	B,%	Number	A,%	B,%	
**Aerobic**							
**Gram-positive**							
*Staphylococcus aureus*							
MRSA	34	30.9	13.8	15	3.9	6.1	49 (19.8)
MSSA	26	23.6	10.5	16	4.2	6.5	42 (16.6)
Coagulase-negative S*taphylococcus*	3	2.7	1.2	13	3.4	5.3	16 (6.5)
*Staphylococcus saprophyticus*				1	0.3	0.4	1 (0.4)
*Streptococcus viridans*	1	0.9	0.4	18	4.7	7.3	19 (7.7)
*Streptococcus*							
Group A *Streptococcus*	6	5.5	2.4	9	2.4	3.6	15 (6.1)
Group B *Streptococcus*	3	2.7	1.2	14	3.7	5.7	17 (6.9)
Group G *Streptococcus*				6	1.6	2.4	6 (2.4)
Other *Streptococcus*				1	0.3	0.4	1 (0.4)
*Enterococcus*	4	3.6	1.6	33	8.7	13.4	37 (15.0)
*Bacillus *spp.				4	1.1	1.6	4 (1.6)
*Corynebacterium spp*.				2	0.5	0.8	2 (0.8)
**Gram-negative**							
*Vibrio *spp.	10	9.1	4.0	4	1.1	1.6	14 (5.7)
*Klebsiella *spp.	6	5.5	2.4	26	6.8	10.5	32 (13.0)
*Proteus *spp.	3	2.7	1.2	21	5.5	8.5	24 (9.7)
*Escherichia coli*	2	1.8	0.8	23	6.1	9.3	25 (10.1)
*Morganella morganii*	1	0.9	0.4	15	3.9	6.1	16 (6.5)
*Enterobacter *spp.	1	0.9	0.4	12	3.2	4.9	13 (5.3)
*Serratia marcescens*	1	0.9	0.4	2	0.5	0.8	3 (1.2)
*Shewanella puterfacines*	1	0.9	0.4	1	0.3	0.4	2 (0.8)
*Pseudmonas aeruginosa*				9	2.4	3.6	9 (3.6)
*Aeromonas *spp.				7	1.8	2.8	7 (2.8)
*Citrobacter *spp.				4	1.1	1.6	4 (1.6)
*Acinetobacter baumannii*				3	0.8	1.2	3 (1.2)
*Stenotroph maltophilia*				2	0.5	0.8	2 (0.8)
*Burkholderia gladioli*				1	0.3	0.4	1 (0.4)
*Haemophilus influenzae*				1	0.3	0.4	1 (0.4)
*Providencia rettger*				1	0.3	0.4	1 (0.4)
**Anaerobic**							
*Bacteroides *spp.	3	2.7	1.2	29	7.6	11.7	32 (13.0)
*Peptostrept *spp.	2	1.8	0.8	35	9.2	14.2	37 (15.0)
*Prevotella *spp.	1	0.9	0.4	31	8.2	12.6	32 (13.0)
*Fusobacterium *spp.	1	0.9	0.4	9	2.4	3.6	10 (4.0)
*Veillonella *spp.				8	2.1	3.2	8 (3.2)
*Clostridium *spp.				2	0.5	0.8	2 (0.8)
*Porphyromonas *spp.				1	0.3	0.4	1 (0.4)
**Fungus**							
*Candida albicans*	1	0.9	0.4	1	0.3	0.4	2 (0.8)
							
**Total**	110	100	44.5	380	100	55.5	247 (100)

*:A = [Number of isolate of the microbial species/Total number of monomicrobial (110) or polymicrobial (380)] × 100%

B = (Number of isolation/247) × 100%

### Management and antimicrobial susceptibility of 49 MRSA infections

All patients were urgently treated with surgical debridement or amputation of the infected part. Generally, the wounds were repaired with delayed closure, split-thickness skin grafts, or flap reconstructive surgery; the remaining small skin defects were allowed to close by secondary intention. Within 24 h after admission, most patients with MRSA infections (41/49, 83.7%) were prescribed empiric antibiotic therapy with β-lactam antimicrobials and gentamicin. No patient with MRSA necrotizing fasciitis was prescribed empiric antibiotic therapy with vancomycin. Vancomycin was subsequently administered to 23 (46.9%) patients after the susceptibility test had been completed. All MRSA isolates were susceptible to linezolid, teicoplanin, and vancomycin, but highly resistant to erythromycin and clindamycin (>90%) and mildly resistant to tetracycline (>65%) and fucidin (12%). Interestingly, resistance to trimethoprim-sulfamethoxazole differed significantly between CA- and HA-MRSA groups (4% *vs*. 45.8%).

### Prevalence of SCC*mec *types and genes for PVL, Hlg, TSST-1, ETA, and ETB

PCR amplification identified five SCC*mec *types, which were SCC*mec *III, IV, V, VT (or VII) and the nontypeable type. The most prevalent SCC*mec *types were SCC*mec *IV in CA-MRSA and SCC*mec *III in HA-MRSA. Isolates that contained the SCC*mec *III element were multi-drug resistant to β-lactams, erythromycin, clindamycin, and trimethoprim-sulfamethoxazole. Unlike a single HA-MRSA isolate that contained the pulsotype C/SCC*mec *IV element, isolates that contained SCC*mec *IV, V, or VII elements were susceptible to trimethoprim-sulfamethoxazole; however, all isolates were resistant to β-lactams, erythromycin, and clindamycin. Among the genes for five virulence factors, those for Hlg were present in all isolates and those for ETA, TSST-1, and PVL were present in a few isolates. Notably, the PVL genes were identified in two CA-MRSA isolates and one HA-MRSA isolate. The ETB gene was not detected in any sample (Table 3).

**Table 3:** Characteristics of 16 CA- and HA-MRSA and their clinical association

Isolate	ST	Allelic profile							PFGE	Pulsotype*	Antibiogram								SCC*mec *	CA/HA	Virulence	Clinical
		*arc*C	*aro*E	*glp*F	*gmk*	*pta*	*tpi*	*yqi*L	genotype		CC	CIP	ERY	FA	GM	KM	SXT	TE	type	MRSA	factor	association
SA141	9	3	3	1	1	1	1	10	gt-7	ND1	R	R	R	S	R	R	S	R	V	HA	Hlg	Healed
SA93	30	2	2	2	2	6	3	2	gt-8	ND2	R	I	R	S	R	R	S	S	IV	CA	Hlg	Bacteremia
SA49	59	19	23	15	2	19	20	15	gt-2a	C	R	I	R	S	R	R	S	S	IV	HA	Hlg, ETA	Amputation
SA52	59	19	23	15	2	19	20	15	gt-2b	C	R	I	R	S	R	R	S	R	IV	CA	Hlg, TSST	Healed
SA104	59	19	23	15	2	19	20	15	gt-2c	C	R	I	R	S	I	I	S	S	IV	HA	Hlg	Healed
SA46	59	19	23	15	2	19	20	15	gt-2d	C	R	I	R	S	I	R	S	S	IV	CA	Hlg, ETA	Amputation
SA10	59	19	23	15	2	19	20	15	gt-2e	C	R	I	R	S	I	R	S	R	IV	HA	Hlg	Healed
SA215	59	19	23	15	2	19	20	15	gt-3	D	R	I	R	S	I	R	S	S	V	HA	Hlg, PVL	Skin graft
SA100	59	19	23	15	2	19	20	15	gt-4	D	R	I	R	S	I	R	R	R	IV	HA	Hlg	Bacteremia, Amputation
SA123	59	19	23	15	2	19	20	15	gt-5a	D	R	I	R	S	I	R	S	R	VII	CA	Hlg, PVL	Healed
SA156	59	19	23	15	2	19	20	15	gt-5b	D	R	I	R	S	I	R	S	S	V	CA	Hlg, PVL	Skin graft
SA19	239	2	3	1	1	4	4	3	gt-1a	A	R	R	R	R	R	R	R	R	III	HA	Hlg	Amputation
SA31	239	2	3	1	1	4	4	3	gt-1b	A	R	R	R	S	R	R	R	R	III	HA	Hlg	Bacteremia
SA151	239	2	3	1	1	4	4	3	gt-1c	A	R	R	R	S	R	R	S	R	III	HA	Hlg	Amputation
SA167	238 & 585 like	2	1a	1	1	4	4	1b	gt-1c	A	R	R	R	S	R	R	S	R	III	HA	Hlg	Bacteremia, amputation
SA165	1279 like	1	3c	1	1	12	1	3	gt-6	O	R	I	R	S	R	R	S	R	Non-type	HA	Hlg	Bacteremia

* ND means that pulsotype could not be determined and pulsotypes were similar to those reported in Huang *et al*. [7].

### PFGE patterns and MLST types

**Figure 2:**
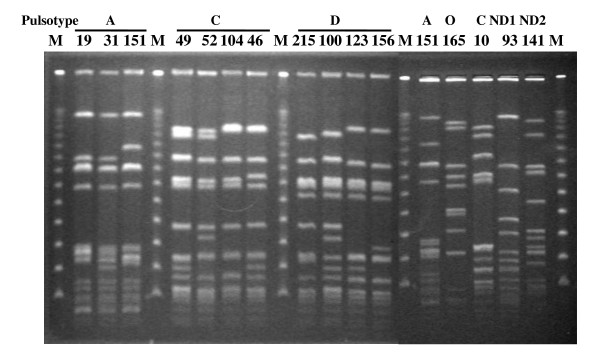
**PFGE patterns of *Sma*I-digested genomic DNA of MRSA isolates collected from the patients with necrotizing fasciitis**. M: lambda DNA. ND1 and ND2 mean new pulsotypes.

Analysis of *Sma*I-digested PFGE identified 16 MRSA isolates associated with seven pulsotypes (Figure 2). In comparison with the resolutions of PFGE patterns described by Huang [7], genotype A, C, and D, the major clones in that report, consisted of 81.3% of the isolates in the present study (Table 3). The pulsotypes were associated with the SCC*mec *types as pulsotype A/SCC*mec *III and pulsotype C/SCC*mec *IV. In the pulsotype D, SCC*mec *types IV, V, and VII were identified and three out of four pulsotype D isolates had the genes for PVL. MLST analysis identified six MLST types in that 75% (12/16) of the isolates were ST59 and ST 239. Additionally, other four types were ST9, ST30, ST1279-like, ST238- and 585-like types (Table 3).

## Discussion

The mainstay treatments of necrotizing fasciitis are urgent operation for decompression and drainage, subsequently repeated debridement, and appropriate antibiotics. The selection of appropriate antimicrobial agents for any suspected necrotizing fasciitis must take into account the nature of patient's exposure and local epidemiologic data. However, there are no reliable epidemiological or clinical risk factors that could distinguish patients infected with MRSA from those infected with MSSA or non-*S. aureus *[33]. With the explosion of MRSA infections, recent reports have established that MRSA is responsible for 3.6% to 39% of NF disease [1,4-6]. In this study, the prevalence of MRSA in 247 NF cases over a four-year period was 19.8% (Table 1 and Figure 1). The variation in the prevalence of MRSA necrotizing fasciitis around the world may reflect variations in genetic background and local epidemiology. MRSA is gradually becoming the main monomicrobial pathogen to cause NF in Taiwan, reflecting changes in the microbial flora in this area. In this investigation, the observed mortality rate did not significantly vary among the four groups, even though NF patients with HA-MRSA infection had a higher incidence of comorbidity on admission than those infected by CA-MRSA, MSSA or non-*S. aureus *group. However, the amputation rate and reconstruction rate for NF patients with MRSA infection were 18.4% and 30.6% respectively - significantly higher than those infected by non-*S. aureus*. These analytical findings further highlight the role of MRSA as an important pathogen in causing a destructive and deep-seated infection.

Recent reports have demonstrated that the CA-MRSA strains with specific virulence factors have a shorter doubling time than HA-MRSA strains and tend to be more virulent [34]. PVL has been associated with epidemic CA-MRSA strains causing lethal necrotizing fasciitis [35]. Contrary to these findings, in the present investigation, all of the patients who were infected by CA-MRSA survived. Miller [4] reported no deaths in their series, hypothesizing that necrotizing fasciitis that is caused by CA-MRSA may be less virulent than similar infections that are caused by other organisms. According to the findings herein, patients who were infected by CA-MRSA tended to have less severe clinical characteristics in terms of comorbidity, amputation rate and the involvement of lower extremities than those with HA-MRSA infections, suggesting that it may be associated with limited elevation of C-reactive protein in patients as well as lower virulence of CA-MRSA strains which differed distinctly from the highly virulent PVL-positive CA-MRSA strains spreading elsewhere.

In the present study, the PVL genes were only identified in two CA- and one HA-MRSA isolates. Genes for the virulence factors TSST-1 and ETA were present only in a few MRSA isolates. However, patients who were infected by pulsotype C isolates with the ETA gene all underwent amputation (Table 3). Unlike the Hlg genes, which were present in all isolates, the ETB gene was absent from all isolates. Other series has also reported a lower frequency of toxin genes carriage. Miller reported that no TSST-1, ETA and ETB genes were identified in any of the CA-MRSA strains [4]. The severity of PVL-specific pathology remains controversial. The PVL genes have been detected in approximately 50% of *S. aureus *isolates that are responsible for subcutaneous abscesses and cellulitis, but not in isolates that are associated with deep-seated infections [36]. A study of isogenic PVL strains of pulsotypes USA300 and USA400 also found that PVL was not the major virulence factor for necrotizing fasciitis [37]. Conversely, the PVL gene has been detected in five NF-associated ST8/pulsotype USA 300/SCC*mec *IV CA-MRSA isolates [4]. A recent investigation stated that PVL tends to damage the muscle rather than skin in young mice [38]. These results are consistent with the clinical characteristics of necrotizing fasciitis, which typically damages deep tissues but may spare skin. Thromboangiitis obliterans followed by the occlusion of cutaneous perforators may be the leading cause of NF cases with extensive skin necrosis - rather than direct cutaneous invasion.

In Taiwan, ST239/pulsotype A type is the most prevalent clone of HA-MRSA and the ST59/pulsotype C and D isolates are the dominant clones of CA-MRSA [30,39-41]. Moreover, SCC*mec *VII and multi-drug resistant CA-MRSA isolates have been identified in pediatric patients with skin and soft tissue infections in northern Taiwan [29]. This investigation identified various NF-associated MRSA strains, which have been the prevalent clones in Taiwan. These strains differ distinctly from the particular ST8/pulsotype USA300 CA-MRSA clone, which has been identified as a dominant cause of necrotizing fasciitis in the USA [4,6]. The results herein verified that the SCC*mec *III type was found only in HA-MRSA and the SCC*mec *IV element was present in both CA- and HA-MRSA isolates with ST59/pulsotype C and pulsotype D (Table 3), suggesting transmission of the community strain of MRSA into the hospital setting, and that the community strain had became a hospital-associated pathogen in Taiwan [9].

Empiric antibiotic therapy for NF patients must be modified for the broad coverage of the regional MRSA strains. Earlier, antimicrobial susceptibility patterns could be used to differentiate CA-MRSA from HA-MRSA isolates; however, this approach is no longer reliable because CA-MRSA may also develop resistance to non-β-lactam antibiotics [34]. Clindamycin has been a common prescribed medication for MRSA infection. In this investigation, both CA- and HA-MRSA isolates exhibited extremely high resistance (>90%) to erythromycin and clindamycin (Table 1), which were contradicting findings obtained from different countries, but supporting other findings from Taiwan [5,7,42]. Trimethoprim-sulfamethoxazole was the only drug associated with different susceptibility between CA- and HA-MRSA strains, to which CA-MRSA isolates were significantly more susceptible than HA-MRSA isolates [9]. For complex skin and soft tissue infections associated with considerable comorbidities, treatment with appropriate antimicrobials is required to reduce clinical failures. Delayed treatments are associated with the morbidity and mortality of MRSA infection [43]. The most common antibiotic prescribing error in this investigation was inadequate coverage of the pathogen MRSA. Even though vancomycin was administered to 46.9% of MRSA patients after the susceptibility test, 83.7% of the patients with MRSA infections herein underwent empiric therapy with antibiotics that were not active against the MRSA strains at the time of hospital admission. This fact may explain the higher amputation and reconstruction rates of patients who were infected by MRSA than those of patients who were infected by MSSA or non-*S. aureus*. An effective empiric antimicrobial therapy may be critical to ensure a favorable surgical outcome for NF patients with MRSA infection. The empiric use of vancomycin should be considered for NF patients based on epidemiologic data and nature of patient's exposure in an area where high prevalence of methicillin resistance was noted such as Taiwan. Additionally, to prevent the emergence of the resistant strains and prolong the effectiveness of currently available antimicrobials, empiric therapy using agents such as vancomycin should not be prescribed for immuno-competent patients with mild skin and soft-tissue infections [44].

## Conclusion

MRSA has emerged as an important cause of necrotizing fasciitis in Taiwan. In this investigation, the prevalence of MRSA in 247 NF cases over a four-year period was 19.8%. NF patients with MRSA infection presented more severe clinical outcomes in terms of amputation rate and reconstruction rate than those with MSSA or non-*S. aureus *infection. In contrast to CA-MRSA infection, NF patients with HA-MRSA infection had a significantly higher amputation rate, comorbidity, C-reactive protein level, and involvement of lower extremity. Further, HA-MRSA isolates were more resistant to trimethoprim-sulfamethoxazole. The ST239/pulsotype A, ST59/pulsotype C and D MRSA are the major pathogenic clones to cause necrotizing fasciitis. The ST59/pulsotype D/SCC*mec *V/PVL MRSA isolates were found in both CA- and HA-MRSA; the ETA gene was found in ST59/pulsotype C/SCC*mec *type IV CA- and HA-MRSA. As well as surgical debridement, initial empiric antimicrobials with a broad coverage for MRSA should be considered in the treatment of NF patients in an endemic area.

## List of abbreviations

CA-MRSA: community-acquired methicillin-resistant *Staphylococcus aureus*; CLSI: Clinical and Laboratory Standards Institute; ETA: exfoliatin A; ETB: exfoliatin B; HA-MRSA: hospital-acquired MRSA; Hlg: γ-hemolysin; MLST: multilocus sequence typing; MSSA: methicillin-susceptible *Staphylococcus aureus*; NF: necrotizing fasciitis; PFGE: pulsed field gel electrophoresis; PVL: Panton-Valentine leukocidin; RA: rifampicin; SCC*mec*: staphylococcal chromosomal cassette *mec*; TSST-1: toxic shock syndrome toxin 1.

## Competing interests

The authors declare that they have no competing interests.

## Authors' contributions

CHC and CC took initiative in developing the research project and manuscript preparation. YYC, SWC, WLC, and JGT participated in the design, laboratory work, and data analysis. All authors read and approved the final manuscript.

## Pre-publication history

The pre-publication history for this paper can be accessed here:

http://www.biomedcentral.com/1471-2334/11/297/prepub
